# N-acetyldopamine dimer inhibits neuroinflammation through the TLR4/NF-κB and NLRP3/Caspase-1 pathways: N-acetyldopamine dimer inhibits neuroinflammation

**DOI:** 10.3724/abbs.2022116

**Published:** 2022-08-26

**Authors:** Lijun Huang, Leiqiang Gong, Xueyan Huo, Lirong Lei, Qi Zhang, Yunjie Hu, Qixuan Kuang, Yu Gui, Yifei Dai, Yucheng Gu, Yun Deng, Dong Wang, Dale Guo

**Affiliations:** 1 State Key Laboratory of Southwestern Chinese Medicine Resources Chengdu University of Traditional Chinese Medicine Chengdu 611137 China; 2 Department of Basic Medical Sciences School of Medicine Tsinghua University Beijing 100084 China; 3 Syngenta Jealott’s Hill International Research Centre Berkshire RG426EY UK

**Keywords:** N-acetyldopamine dimer, neuroinflammation, TLR4/NF-κB pathway, NLRP3/Caspase-1 pathway, surface plasmon resonance assay

## Abstract

Neuroinflammation mediated by microglia is an important pathophysiological mechanism in neurodegenerative diseases. However, there is a lack of effective drugs to treat neuroinflammation. N-acetyldopamine dimer (NADD) is a natural compound from the traditional Chinese medicine *Isaria cicada*. In our previous study, we found that NADD can attenuate DSS-induced ulcerative colitis by suppressing the NF-κB and MAPK pathways. Does NADD inhibit neuroinflammation, and what is the target of NADD? To answer this question, lipopolysaccharide (LPS)-stimulated BV-2 microglia was used as a cell model to investigate the effect of NADD on neuroinflammation. Nitric oxide (NO) detection, reactive oxygen species (ROS) detection and enzyme-linked immunosorbent assay (ELISA) results show that NADD attenuates inflammatory signals and proinflammatory cytokines in LPS-stimulated BV-2 microglia, including NO, ROS, tumor necrosis factor (TNF)-α, interleukin (IL)-1β and interleukin-6 (IL-6). Western blot analysis show that NADD inhibits the protein levels of Toll-like receptor 4 (TLR4), nuclear factor kappa-B (NF-κB), NOD-like receptor thermal protein domain associated protein 3 (NLRP3), ASC and cysteinyl aspartate specific proteinase (Caspase)-1, indicating that NADD may inhibit neuroinflammation through the TLR4/NF-κB and NLRP3/Caspase-1 signaling pathways. In addition, surface plasmon resonance assays and molecular docking demonstrate that NADD binds with TLR4 directly. Our study reveals a new role of NADD in inhibiting the TLR4/NF-κB and NLRP3/Caspase-1 pathways, and shows that TLR4-MD2 is the direct target of NADD, which may provide a potential therapeutic candidate for the treatment of neuroinflammation.

## Introduction

Neuroinflammation generally refers to the immune response of immune cells within the central nervous system (CNS) that can be caused by trauma, ischemia, infection, toxins, autoimmunity and so on [1]. Innate immune cells of the CNS mainly include microglia and astrocytes [2], of which microglia are considered to be the major cells involved in neuroinflammation. Microglia are resident CNS cells that comprise 10% of the CNS population. In addition, microglia execute immune surveillance and perform macrophage-like activities, including the production of proinflammatory factors, such as interleukin (IL)-1β, interleukin-6 (IL-6) and tumor necrosis factor (TNF)-α, chemokines, small molecule messengers, such as nitric oxide, and reactive oxygen species (ROS) [ 1, 3, 4] . Uncontrolled and overactivated microglia can lead to pathological changes in the CNS and further lead to neurodegenerative diseases, such as Alzheimer’s disease and Parkinson’s disease [ 5‒ 8] . Nonetheless, there are no effective drugs to cure neuroinflammatory-associated neurodegenerative diseases.

Toll like receptor 4 (TLR4) belongs to pattern recognition receptors (PRRs), which is a canonical receptor for lipopolysaccharide (LPS) from Gram-negative bacteria. TLR4 can be activated by various CNS stimuli, such as pathogens, different cytokines or even stress [ 9‒ 11] . TLR4 activation contributes to microglial activation and potentiates inflammatory cascade pathways, such as NF-κB pathway and its down-stream’s NLRP3/Caspase-1 pathway [ 12‒ 14] . Over-activation of the TLR4/NF-κB pathway in microglia usually leads to serious immune response by inducing the release of proinflammatory cytokines, chemokines and ROS [15]. Thus, it is proposed that TLR4 may serve as a potential therapeutic pharmacophore for the discovery of natural compounds for the treatment of neurodegenerative disorders associated with neuroinflammation [11].

N-acetyldopamine dimer (NADD) is a natural compound and has been elucidated as (2R,3S)-2-(3′,4′-dihydroxyphenyl)-3- acetylamino-7-(N-acetyl-2″-aminoethyl)-1,4-benzodioxane. In our previous study, NADD extracted from the traditional Chinese medicine *Isaria cicada* was found to suppress inflammation in DSS-induced ulcerative colitis by inhibiting the NF-κB and MAPK signaling pathways [16]. In addition, NADD has been reported to possess antioxidant and anti-inflammatory activities [17]. However, whether NADD inhibits neuroinflammation is still unknown.

In the present study, we investigated the suppressive effects of NADD on neuroinflammation and clarified the underlying mechanisms utilizing the lipopolysaccharide (LPS)-activated BV-2 microglial cell inflammatory model.

## Materials and Methods

### Cell culture

BV-2 microglial cells (gifted by Lixia Qin, Chengdu University of Traditional Chinese Medicine, Chengdu, China) were cultured in Dulbecco’s modified Eagle’s medium (DMEM; Gibco, Carlsbad, USA) supplemented with 10% Fetal bovine serum (FBS; Gemini, Calabasas, USA), 100 units/mL penicillin and 100 μg/mL streptomycin (HyClone, Logan, USA) in a humidified atmosphere with 5% CO _2_ at 37°C. Cells were digested with 0.25% trypsin (Servicebio, Wuhan, China) when they were passaged or seeded into plates. NADD and LPS (Beyotime, Shanghai, China) were dissolved in dimethyl sulfoxide (DMSO; Solarbio, Beijing, China). Before treatment, cells were starved for 6 h in FBS-free DMEM. Then, BV-2 microglial cells were pretreated with NADD for 1 h, followed by LPS stimulation for 24 h, and DMSO was used as a control.

### Cell viability assay

BV-2 microglial cells were seeded into 96-well plates at 8×10 ^3^ cells per well and cultured overnight. When cell confluency reached approximately 60%, cells were pretreated with 0, 10, 30, 60, 100, and 200 μM NADD for 1 h and then co-treated with or without 1 μg/mL LPS for 24 h. Then, 10 μL of Cell Counting Kit-8 (CCK8) reagent (MCE, Monmouth Junction, USA) was added to each well and incubated in an incubator for 4 h. Finally, the absorbance of each well was measured at 450 nm with a microplate reader.

### Cell morphological analysis

BV-2 microglial cells were seeded into 6-well plates. When cell confluency reached approximately 60%, cells were starved for 6 h and then pretreated with low (15 μM), middle (30 μM) and high (60 μM) concentrations of NADD for 1 h, followed by co-treatment with or without 1 μg/mL LPS for 24 h. After that, the morphology of each group was observed under an optical microscope (SOPTOP ICX41; Pooher, Shanghai, China).

### Nitric oxide (NO) detection

BV-2 microglial cells were seeded into 6-well plates. When cell confluency reached approximately 60%, cells were starved for 6 h and then pretreated with low (15 μM), middle (30 μM) and high (60 μM) concentrations of NADD for 1 h, followed by co-treatment with or without 1 μg/mL LPS for 24 h. After that, the cell supernatant was collected to detect the production of NO in each group using the NO assay kit (Beyotime) according to the manufacturer’s instructions.

### ELISA assay

BV-2 microglial cells were seeded into 6-well plates. When cell confluency reached approximately 60%, cells were starved for 6 h and then pretreated with low (15 μM), middle (30 μM) and high (60 μM) concentrations of NADD for 1 h, followed by co-treatment with or without 1 μg/mL LPS for 24 h. After that, the cell supernatant was collected to detect the production of IL-6 and TNF-α in each group using the mouse IL-6 ELISA kit and mouse TNF-α ELISA kit (Boster, Wuhan, China) respectively according to the manufacturer’s instructions.

### Detection of reactive oxygen species (ROS)

*In vitro*, BV-2 microglial cells were seeded into 6-well plates. When cell confluency reached approximately 60%, cells were starved for 6 h and then pretreated with low (15 μM), middle (30 μM) and high (60 μM) concentrations of NADD for 1 h, followed by co-treatment with or without 1 μg/mL LPS for 24 h. Then, the generation of ROS in BV-2 microglial cells was detected using the DCFH-DA kit (Ueland, Suzhou, China) according to the manufacturer’s instructions. The DCF produced from DCFH-DA by ROS oxidization in cells was observed under a fluorescence microscope.

*In vivo*, zebrafish embryos were pretreated with low (15 μM), middle (30 μM) and high (60 μM) concentrations of NADD for 1 h and then co-treated with or without 10 μg/mL LPS for 72 h. During this period, NADD and LPS were refreshed every 24 h. Then, the zebrafish were treated with DCFH-DA for 1 h and anesthetized with tricaine as previous study [18]. Finally, zebrafish were observed under a laser confocal microscope (Olympus, Tokyo, Japan). The use and care of animals were approved by the Ethics Committee of Chengdu University of Traditional Chinese Medicine and carried out in strict accordance with the recommendations in the Guide for the Care and Use of Laboratory Animals of the National Institutes of Health.

### Nuclear translocation of NF-κB

BV-2 microglial cells were seeded into 24-well plates. When cell confluency reached approximately 60%, cells were starved for 6 h and then pretreated with low (15 μM), middle (30 μM) and high (60 μM) concentrations of NADD for 1 h, followed by co-treatment with or without 1 μg/mL LPS for 2 h. Then, the nuclear translocation of NF-κB in the cells was detected using the NF-κB activation, nuclear translocation assay kit (Beyotime) according to the manufacturer’s instructions. In brief, cells were fixed with fixing solution for 15 min at room temperature. After the cells were washed, they were blocked with blocking buffer and then incubated with NF-κB/P65 antibody overnight at 4°C. After washing, Cy3-conjugated anti-rabbit IgG antibody was added and incubated at room temperature for 1 h. Consequently, DAPI was added to stain the nuclei for 5 min at room temperature. Finally, the cells were observed under a fluorescence microscope (Olympus).

### Real-time reverse transcription polymerase chain reaction (RT-qPCR)

BV-2 microglial cells were seeded into 6-well plates. When cell confluency reached approximately 60%, cells were starved for 6 h and then pretreated with low (15 μM), middle (30 μM) and high (60 μM) concentrations of NADD for 1 h, followed by co-treatment with or without 1 μg/mL LPS for 24 h. After that, the cells were lysed using the RNA isolater total RNA extraction reagent (Vazyme, Nanjing, China) and total RNA was extracted according to the manufacturer’s instructions. Subsequently, RNA was reverse transcribed into cDNA using an RT EasyTM II kit (Forgene, Chengdu, China), and the cDNA was used as a template to perform fluorescence quantitative PCR using a Real-time PCR EasyTM-SYBR Green I kit (Forgene). All the protocols were performed according to the manufacturer’s instructions. The mRNA levels were calculated as previously described [19]. The primers used are: *IL-1β* forward 5′-TGAAATGCCACCTTTTGACAG-3′, reverse 5′-CCACAGCCACAATGAGTGATAC-3′; *iNOS* forward 5′-GAGCCACAGTCCTCTTTGCTA-3′, reverse 5′-TGTCACCACCAGCAGTAGTTG-3′; and *β-actin* forward 5′-ACCCACACTGTGCCCATCTA-3′, reverse 5′-CACGCTCGGTCAGGATCTTC-3′. *β-Actin* was used as reference gene, the relative expressions of *IL-1β* and *iNOS* versus *β-actin* were quantified.

### Western blot analysis

BV-2 microglial cells were seeded into 6-well plates. When cell confluency reached approximately 60%, cells were starved for 6 h and then pretreated with low (15 μM), middle (30 μM) and high (60 μM) concentrations of NADD for 1 h, followed by co-treatment with or without 1 μg/mL LPS for 24 h. After that, the cells were lysed with 1×SDS lysis buffer and boiled for 30 min [20]. Then, the protein concentration of each group was detected using a BCA Protein Assay Kit (Beyotime) according to the manufacturer’s instructions. After that, equal amounts of protein from each group were subjected to sodium dodecyl sulfate–polyacrylamide gel electrophoresis (SDS–PAGE) and were transferred to polyvinylidene fluoride (PVDF) membranes, followed by blocking for 1 h at room temperature with QuickBlock™ Western blocking buffer (Beyotime). Subsequently, the PVDF membranes were incubated with primary antibodies overnight at 4°C. The primary antibodies were as follows: TLR4 (1:4000; Proteintech, Rosemont, USA), NF-κB (1:1000; CST, Beverly, USA), NLRP3 (1:1000; CST), Caspase-1 (1:1000; Proteintech), ASC/TMS1 (1:1000; Proteintech), iNOS (1:1000; NOVUS, Littleton, USA), COX-2 (1:1000; Abcam, Cambridge, UK), and β-Actin (1:20,000; Proteintech). Then, the PVDF membranes were incubated with the corresponding HRP-conjugated secondary antibodies at room temperature for 2 h. Finally, the membranes were washed 3 times with PBS, the target proteins were visualized using Immobilon western chemiluminescent HRP substrate (Millipore, Billerica, USA), and the blot images were captured and analyzed with the Chemiluminescent Imaging System (SAGECREATION, Beijing, China ) [16].

### Surface plasmon resonance (SPR) analysis

TLR4-MD2 protein (Sino Biological, Beijing, China) was dissolved in sodium acetate buffer (pH 4.5) at 25 μg/mL and immobilized on the CM5 sensor chip (GE Healthcare, Pittsburgh, USA) with an amine-coupling method according to the manufacturer’s protocol. NADD was dissolved in a phosphate buffered solution (PBS) containing 5% DMSO and injected into a TLR4-MD2 protein sensor surface, with the association time, dissociation time and flow rate setting at 180 s, 200 s and 30 μL/min respectively. SPR analyses were performed on Biacore T200 (GE Healthcare).

### Molecular docking

Crystal structures of the TLR4-MD2 complex were obtained from the RCSB Protein Data Bank (PDB ID: 2Z66) [21]. Then, the best available resolution of the crystal structure was chosen. Docking analysis for NADD with TLR4-MD2 was performed using Schrödinger software (Schrödinger, New York, USA). The protein crystal structure, 3D structures, regenerative state of native ligands, H-bond assignment optimization, energy minimization of NADD and water removal were obtained by Schrodinger’s Maestro Molecular modeling suit (Schrödinger). Finally, the Glide SP module was utilized for molecular interactions, according to the known ligand interactions diagram module [22].

### Statistical analysis

All experiments were repeated three times, and data analyses were performed by IBM SPSS Statistics 26 (IBM, New York, USA) using one-way analysis of variance. Data are presented as the mean± standard deviation (SD). A *P*-value less than 0.05 was considered statistically significant.

## Results

### NADD inhibits the inflammation stimulated by LPS

**Figure 1:**
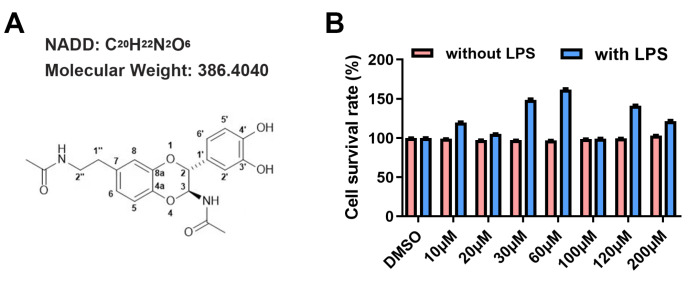
Figure 1 The structure and toxicity of NADD (A) The structure of NADD. (B) Toxicity of NADD measured by CCK8 assay. Red bars indicated that NADD had no toxicity to BV-2 microglial cells which were not stimulated by LPS; blue bars indicated that NADD had no toxicity to BV-2 microglial cells which were activated by LPS as well.

**Figure 2:**
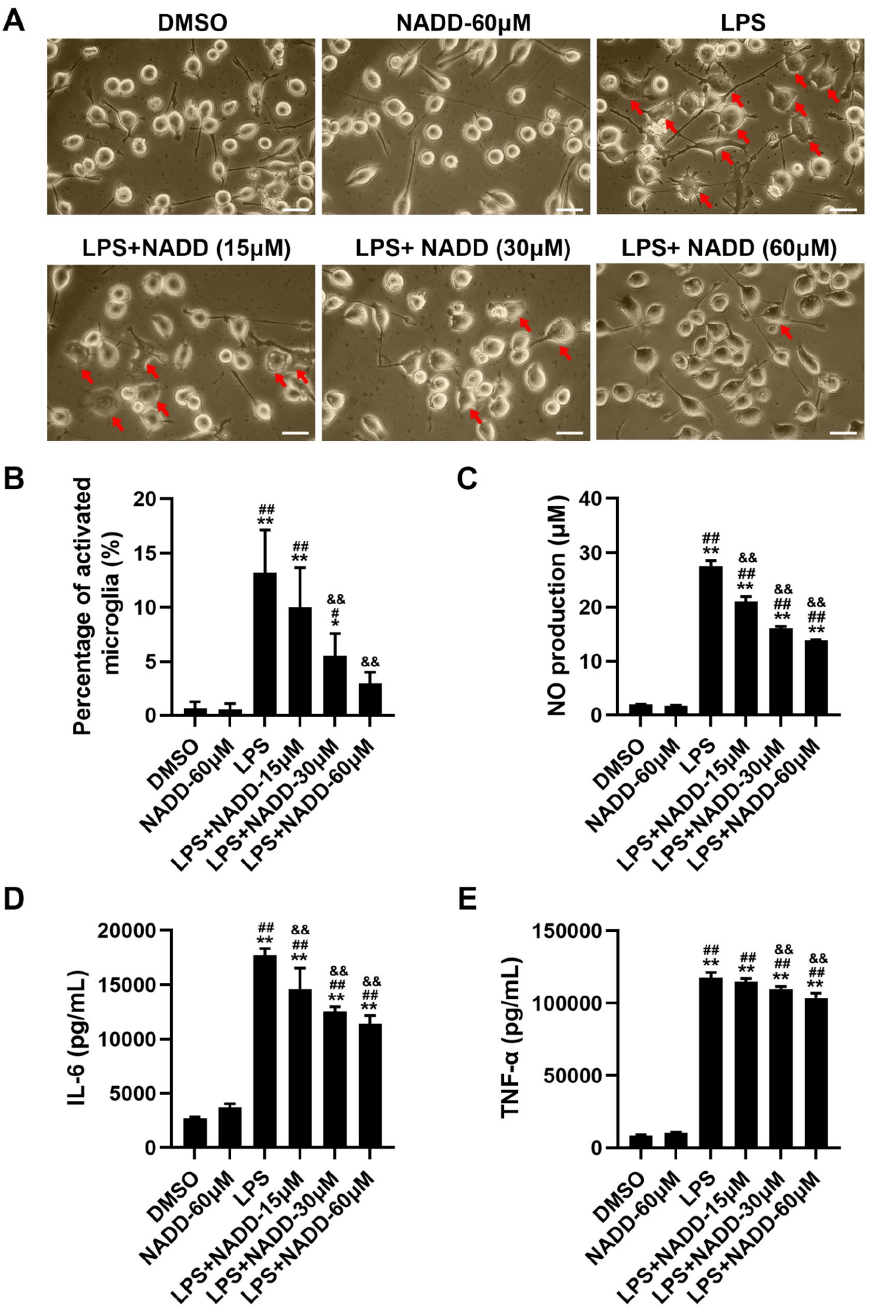
Figure 2 NADD suppresses neuroinflammation (A) The morphology of BV-2 microglial cells pretreated with different concentrations of NADD for 1 h and then co-treated with or without LPS for 24 h; red arrows indicated the activated microglial cells; Scale bar= 100 μm. (B) The statistical analysis of the percentage of morphologic changes in BV-2 microglial cells. (C) Statistical analysis of NO production in cell supernatants of BV-2 cells. (D,E) ELISA results of IL-6 (D) and TNF-α (E) in cell supernatants of BV-2 cells under different treatments. * P<0.05, ** P<0.01 compared with the DMSO group, # P<0.05, ## P<0.01 compared with the NADD -60 μM group, and && P<0.01 compared with the LPS group.

NADD was extracted from *I*. *cicada* as described in our previous study ( Figure 1A). After BV-2 microglial cells were treated with different concentrations of NADD combined with or without LPS for 24 h, CCK8 assay was performed, morphologies of cells were examined by microscopy, and the cell supernatants was collected to measure the production of NO and proinflammatory factors. The results showed that NADD had no inhibitory effects on cell viability regardless of whether BV-2 cells were activated by LPS ( Figure 1B), and it inhibited the activation of BV-2 cells in a concentration-dependent manner ( Figure 2A,B). NADD inhibited the production of NO in a concentration-dependent manner ( Figure 2C). In addition, NADD suppressed the production of IL-6 and TNF-α in a concentration-dependent manner ( Figure 2D,E). These results indicated that NADD could inhibit the production of small proinflammatory factors.

### NADD suppresses the production or expression of inflammatory mediators

**Figure 3:**
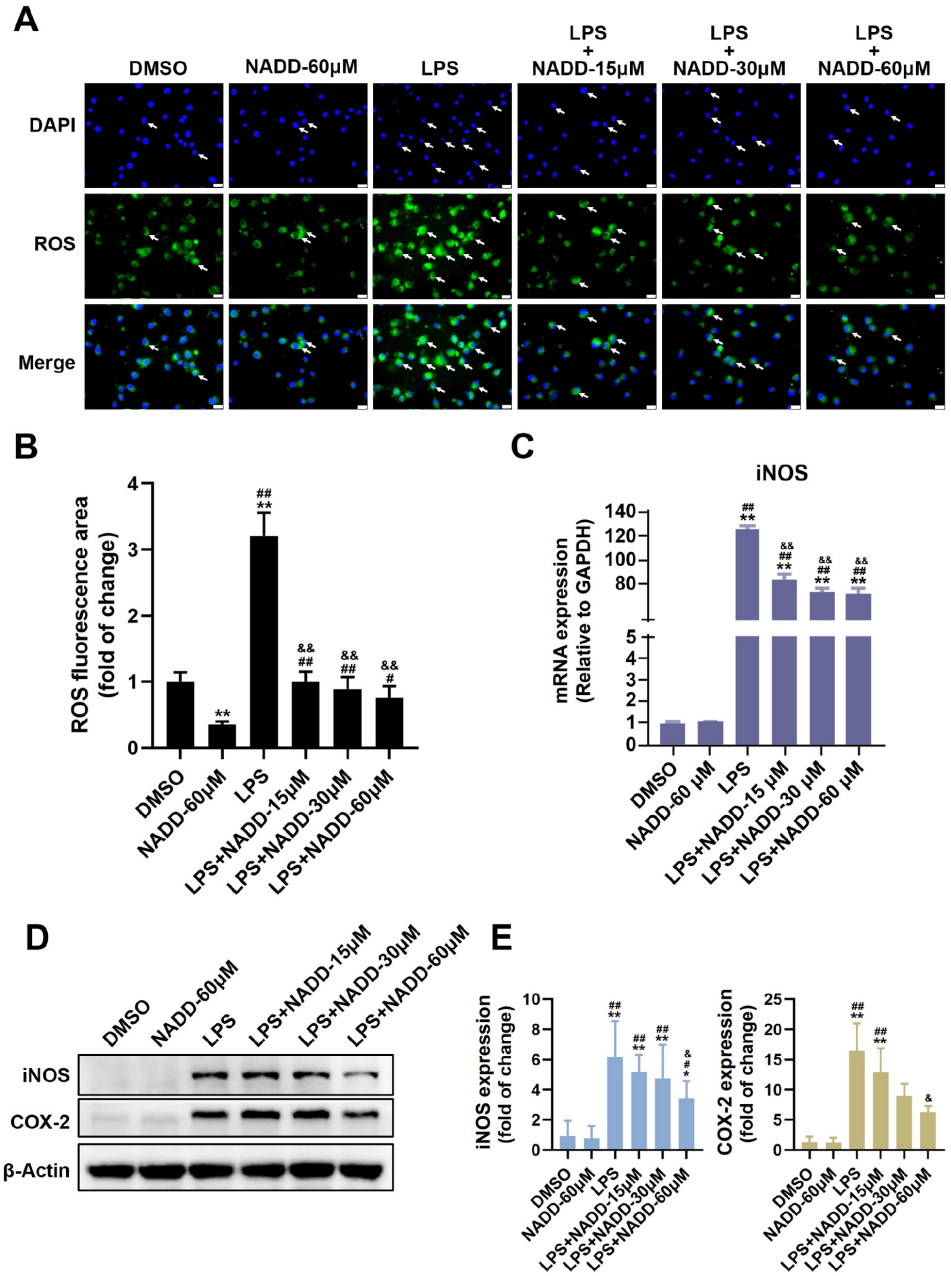
Figure 3 NADD inhibits inflammatory mediators (A) The generation of ROS in BV-2 microglial cells pretreated with different concentrations of NADD for 1 h and then co-treated with or without LPS for 24 h. White arrows indicated the DCF produced from DCFH-DA by ROS oxidization in microglial cells. Scale bar= 50 μm. (B) The statistical analysis of the fluorescence area that indicated the generation of ROS in BV-2 microglial cells. (C) Statistical analysis of transcriptional level of iNOS. (D) Protein expression changes of iNOS and COX-2 in BV-2 cells under different treatments. β-Actin was used as the internal reference. (E) Statistical analysis of expressions of iNOS and COX-2. * P<0.05, ** P<0.01 compared with the DMSO group, # P<0.05, ## P<0.01 compared with the NADD -60 μM group, and & P<0.05, && P<0.01 compared with the LPS group.

It is well known that cyclooxygenase-2 (COX-2) and nitric oxide synthase (iNOS) are two major inflammatory mediators. COX-2 regulates the production of prostaglandin [23], and iNOS induces the generation of NO [24]. In addition, ROS is also an important factor involved in the inflammatory process and may cause endothelial dysfunction and tissue injury [25]. To investigate the effects of NADD on these mediators, we detected the levels of ROS, iNOS and COX-2. Following NADD treatment, ROS generation was decreased in a dose-dependent manner compared with that in the LPS group ( Figure 3A,B). RT-qPCR results showed that NADD downregulated the mRNA level of iNOS with increasing concentrations of NADD ( Figure 3C). Western blot analysis showed that NADD significantly decreased the protein levels of iNOS and COX-2 in a dose-dependent manner ( Figure 3D,E).

### NADD attenuates inflammation *in vivo*

**Figure 4:**
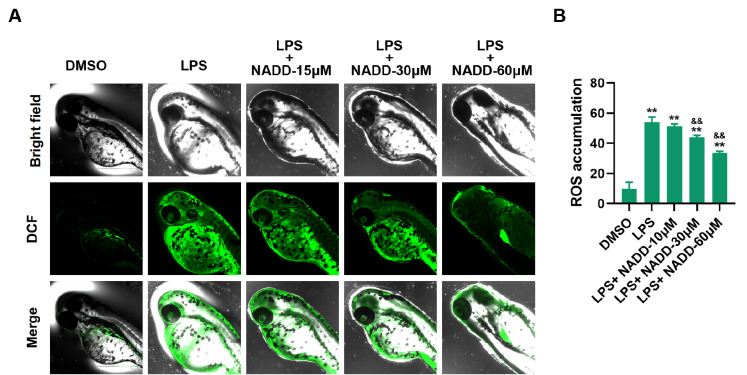
Figure 4 NADD inhibits the generation of ROS in zebrafish (A) Representative image of zebrafish that produce ROS (DCF+, green) in different treatment groups. (B) Fluorescence intensity of DCF was quantified by ImageJ and statistically analyzed. ** P<0.01 compared with the DMSO group, and && P<0.01 compared with the LPS group.

Zebrafish are valuable vertebrate models for bioactivity and safety studies of natural products because they are genetically, anatomically and physiologically similar to humans, breed quickly and are of low cost [ 26, 27] . LPS-induced zebrafish inflammation models have been widely utilized to evaluate the anti-inflammatory properties of natural products in recent years by detecting changes in ROS [27]. In this study, we detected the generation of ROS in LPS-stimulated zebrafish. The results showed that NADD treatment significantly reduced the green fluorescence signals of ROS in a dose-dependent manner ( Figure 4A,B). These results were consistent with the measurements of ROS *in vitro*.

### NADD inhibits the TLR4/NF-κB pathway

**Figure 5:**
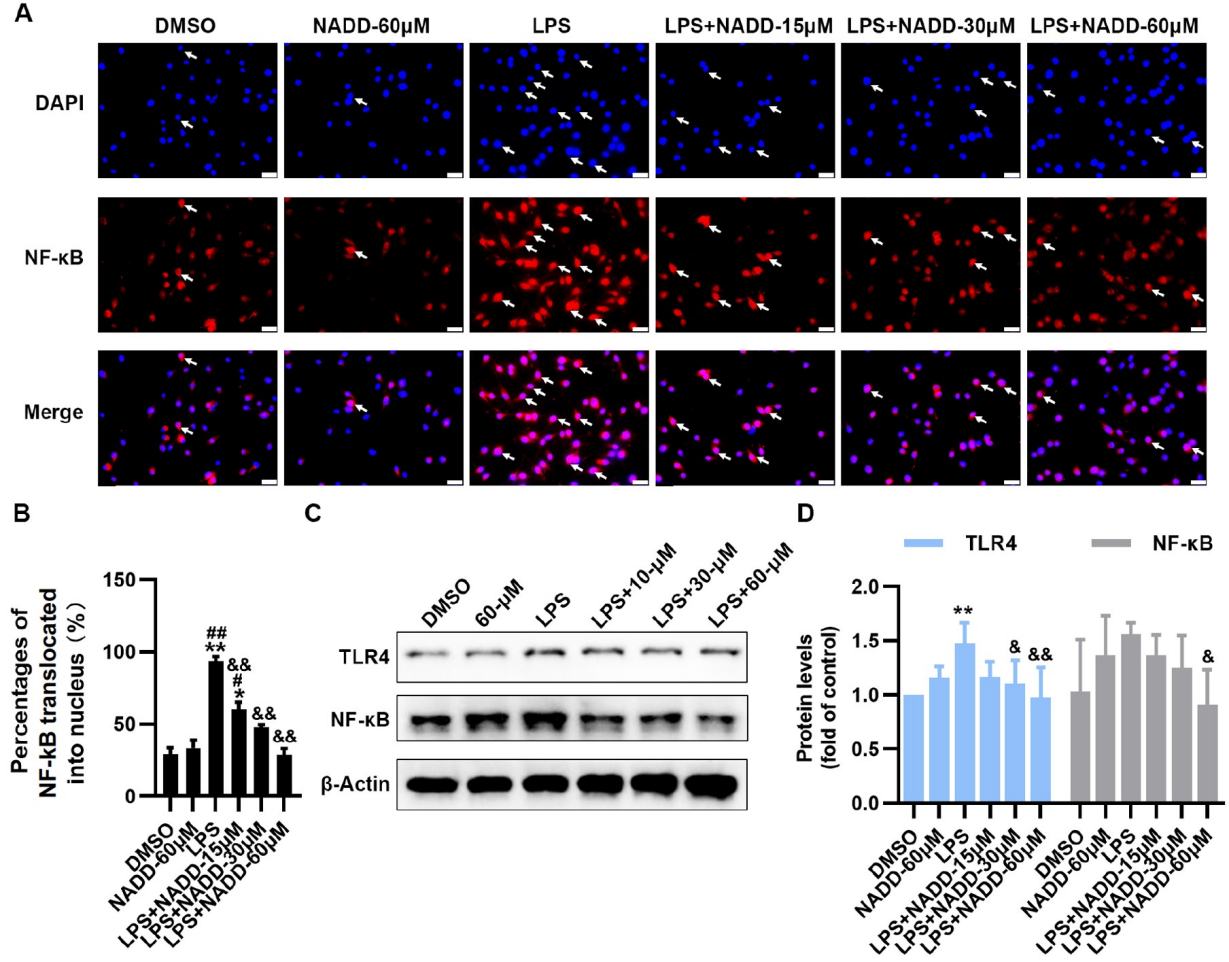
Figure 5 NADD inhibits TLR4/NF-κB pathway in LPS-stimulated BV-2 microglial cells (A) Nuclear translocation of NF-κB in BV-2 microglia pretreated with different concentrations of NADD for 1 h and then co-treated with or without LPS for 2 h. White arrows indicated NF-κB that had been translocated into cell nucleus. Scale bar= 50 μm. (B) The statistical analysis of the percentage of cells in which NF-κB was translocated into the nucleus. (C) Protein expression changes of TLR4 and NF-κB in BV-2 cells under different treatments. β-Actin was used as the internal reference. (D) Statistical analysis of the expressions of TLR4 and NF-κB. * P<0.05, ** P<0.01 compared with the DMSO group, #P<0.05, ## P<0.01 compared with the NADD –60 μM group, and & P<0.05, && P<0.01 compared with the LPS group.

Microglial surfaces express the TLR4 receptor widely, and TLR4 plays a crucial role in mediating inflammation [28]. When the organism is invaded by pathogens and endogenous harmful stimuli, the TLR4 receptor will recognize these stimuli and further trigger the activation of downstream NF-κB [29]. Activated NF-κB then translocates into the nucleus and functions as a transcription factor to increase the generation of proinflammatory molecules [ 18, 30] . To investigate whether NADD affects the TLR4/NF-κB pathway, we detected the translocation of NF-κB and the protein levels of TLR4 and NF-κB in BV-2 cells following the administration of NADD. The results showed that NADD significantly suppressed the activation of NF-κB in a dose-dependent manner ( Figure 5A,B). In addition, the western blot analysis results showed that NADD inhibited the protein expressions of TLR4 and NF-κB compared with that in the LPS group ( Figure 5C,D).

### NADD suppresses the NLRP3/Caspase-1 pathway

**Figure 6:**
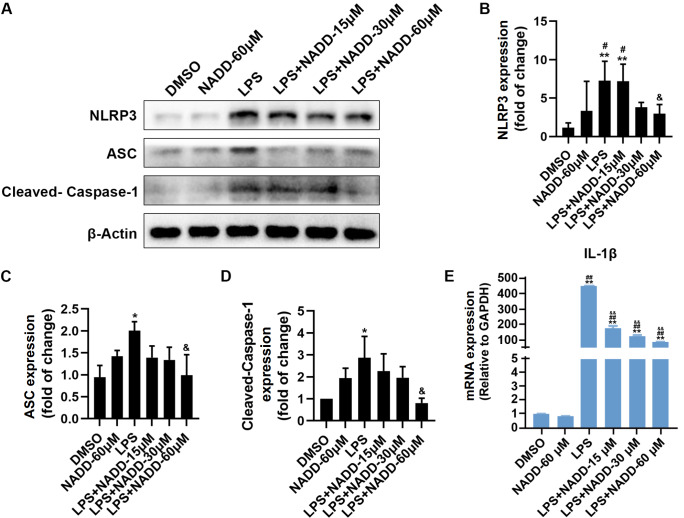
Figure 6 Protein levels of NLRP3/Caspase-1 pathway factors increased by LPSare suppressed by NADD treatment (A) Protein expression changes of NLRP3, ASC and Cleaved-Caspase-1 in BV-2 cells under different treatments. β-Actin was used as the internal reference. (B) Statistical analysis of expression of NLRP3. (C) Statistical analysis of expression of ASC. (D) Statistical analysis of expression of cleaved-Caspase-1. (E) mRNA expression level of IL-1β in different treatment groups. * P<0.05, ** P<0.01 compared with the DMSO group, # P< 0.05, ## P<0.01 compared with the NADD -60 μM group, and & P<0.05, && P<0.01 compared with the LPS group.

The NOD-like receptor (NLR) family pyrin domain–containing protein 3 (NLRP3) inflammasome is an important component of the inflammatory process and is involved in various inflammatory disorders, such as Alzheimer’s disease, multiple sclerosis, type 2 diabetes, and obesity [ 31, 32] . To investigate the effects of NADD on the NLRP3/Caspase-1 pathway, we examined the changes in proteins in this pathway by western blot analysis and RT–PCR. The results showed that LPS significantly increased the protein expressions of NLRP3, ASC and cleaved-Caspase-1 ( Figure 6A‒D) and increased the mRNA level of IL-1β ( Figure 6E). However, when BV-2 microglial cells were treated with NADD for 24 h, the protein levels of NLRP3, ASC and cleaved-Caspase-1were decreased in a concentration-dependent manner ( Figure 6A‒D), and the mRNA level of IL-1β was also decreased in a dose-dependent manner ( Figure 6E). These results suggested that NADD might suppress neuroinflammation through the NLRP3/Caspase-1 pathway.

### NDAA shows affinity for the TLR4-MD2 complex

**Figure 7:**
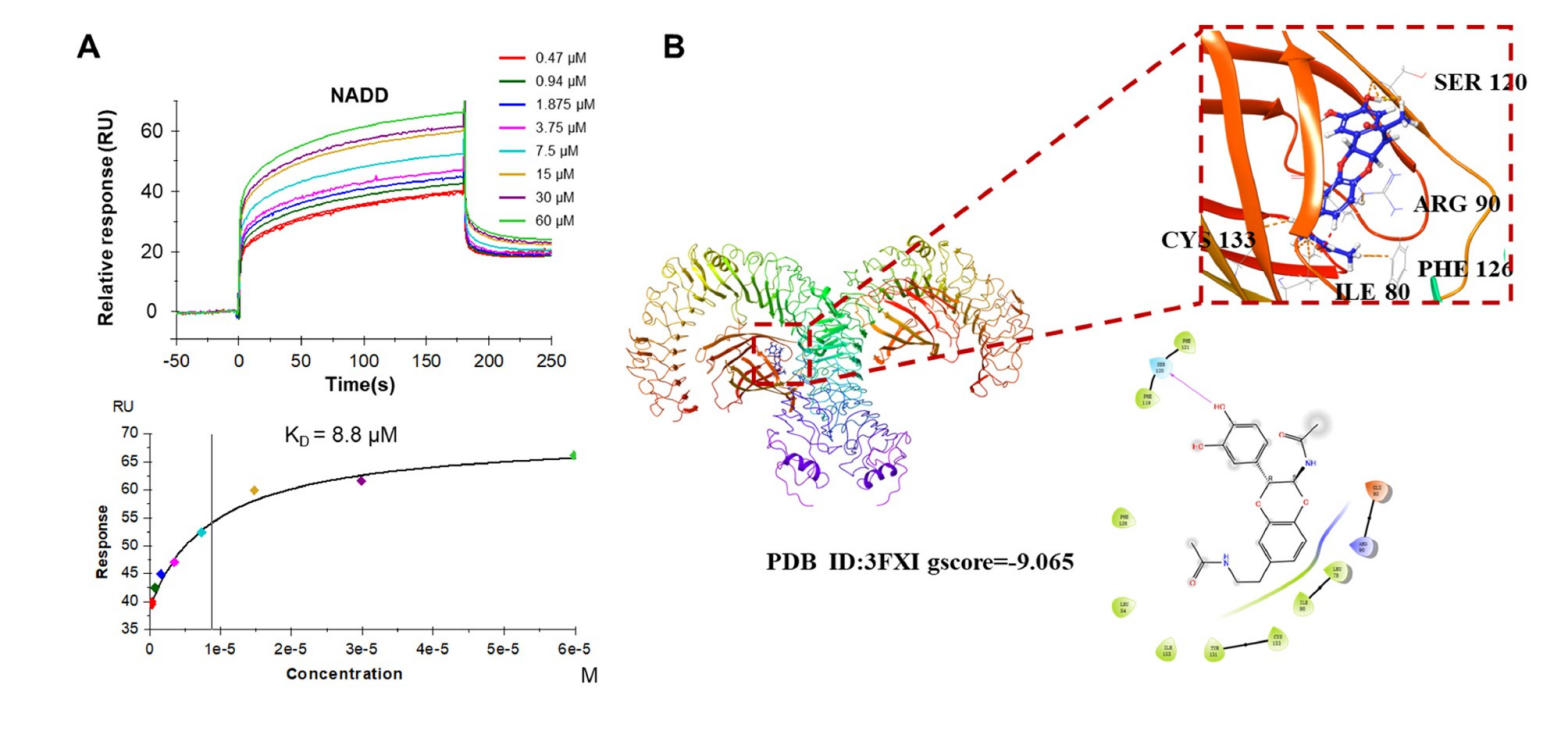
Figure 7 NADD binds to TLR4 directly (A) SPR analysis of the binding affinity of NADD with TLR4-MD2 protein with a KD value of 8.8 μM. Apparent equilibrium dissociation constants (KD) were calculated by global fitting using a steady-state affinity model in the Biacore T200 evaluation software. (B) Molecular docking results of NADD with TLR4-MD2. Molecular docking simulations were obtained at the lowest energy conformation. Hydrogen bonding interactions are shown by dashes.

Considering the important role of the LPS receptor-TLR4 in inflammation both *in vitro* and *in vivo*, we explored whether NADD could compete with LPS in binding to the TLR4-MD2 complex by SPR assay and molecular docking analysis. The SPR results showed that NDAA exhibited affinity for the TLR4-MD2 protein complex with a KD value of 8.8 μM ( Figure 7A). In addition, the molecular docking analysis demonstrated that NADD could dock with the pocket of TLR4-MD2 and interact with multiple amino acids, including ILE-80, ARG-90, SER-120, CYS-133 and PHE-126, which suggested that NADD could bind directly with the TLR4-MD2 complexe and prevent the MD2 protein from binding with the TLR4 receptor, supporting the results of the SPR assay ( Figure 7B). These data suggested that NADD might inhibit inflammation by competing with LPS in binding to the TLR4-MD2 complex, which further prevented LPS from working by binding to MD2.

## Discussion

In this study, we investigated the anti-neuroinflammatory role of NADD in microglia and zebrafish. We demonstrated that NADD may suppress the production of inflammatory message molecules and proinflammatory factors by inhibiting the TLR4/NF-κB and NLRP3/Caspase-1 pathways. Furthermore, we provided evidence that NADD can directly bind with TLR4-MD2, verifying that TLR4-MD2 is the target of NADD.

Studies in neurodegenerative diseases have shown that their mechanisms are poorly understood and there is no cure for them, but neuroinflammation is a key contributor to their pathological processes [33]. Therefore, it is necessary to find candidate small molecule compounds for neurodegenerative diseases in neuroinflammatory models. LPS is an outer membrane component of Gram-negative bacteria and an important tool for the induction of inflammation [33]. The LPS-stimulated BV-2 microglial cell model is a common neuroinflammatory model *in vitro*. Microglial cells are innate immune cells of the CNS and they play a key role in inflammatory responses in the CNS [ 2, 34] . In addition, microglia express the receptors of LPS, such as TLRs [28]. When microglial cells are stimulated by LPS, LPS activates its receptors and further activates its downstream pathways, which ultimately results in neuroinflammation, including an amoeboid morphology of microglia and upregulated production and release of proinflammatory cytokines and cell adhesion molecules, such as IL-1β, IL-6, TNF-α, NO and ROS [ 35‒ 38] . In addition, upon activation, TLR expression may also be enhanced in the microglia [ 39, 40] .

*I*. *cicada* is a kind of traditional Chinese medicine that possesses the same effect as Periostracum cicadae in the description of the “Compendium of Materia Medica”, which has been used as an antifebrile, spasmolytic, sedative and antiphlogistic for over thousands of years [17]. *I*. *cicada* possesses the biological functions of anti-inflammation, antioxidation, antiaging, antitumor, anticonvulsant, immunoregulation, and neuroprotection [ 16, 41‒ 43] . *I*. *cicada* has effects on both the nervous system and inflammation. Many compounds extracted from *I*. *cicada* or *Periostracum cicadae* have anti-inflammatory and antioxidation activities. For example, N6-(2-hydroxyethyl) adenosine and N-acetyldopamine dimer have anti-inflammatory functions [ 16, 17, 44] and adenosine shows antioxidative effects [45]. In this study, we found that NADD extracted from *I*. *cicada* significantly inhibited the neuroinflammation stimulated by LPS, including the alleviation of morphological activation ( Figure 2A), the reduction of proinflammatory factors ( Figure 2D,E), NO ( Figure 2C) and ROS ( Figures 3A,B and 4A,B).

It has been reported that TLR4 can mediate inflammatory neurodegeneration [46], and TLR4 on the microglia may play an important role in inflammation in the CNS [40]. In exploring the mechanism by which NADD inhibits neuroinflammation, we focused on the LPS receptor TLR4. The SPR results showed that NADD showed affinity for the TLR4-MD2 protein complex ( Figure 7A), and molecular docking showed that NADD could directly bind with TLR4-MD2 ( Figure 7B), which suggested that TLR4-MD2 may be the target of NADD. The ligation of TLR4-MD2 stimulated by LPS recruits the adaptors of TRAM, TRIF, TIRAP and MyD88. Subsequently, various kinases and ubiquitin ligases are recruited and activated, consequently initiating the NF-κB, MAPK, and type I interferon pathways [ 37, 47] . The activation of NF-κB promotes the production of inflammatory cytokines, such as IL-1β, upregulates the pyrin domain-containing 3 (NLRP3) inflammasome, and further causes inflammasome assembly by simulating a conformational change in NLRP3 or by TRPM2-mediated calcium influx [ 14, 48] . The activation of NLRP3 facilitates the recruitment of an apoptosis-associated speck-like protein containing a C-terminal caspase recruitment domain (ASC), and then caspase-1 is recruited to the inflammasome and activated. Subsequently, activated caspase-1 facilitates the proteolytic cleavage of ProIL-1β, which produces mature IL-1β [14]. IL-1β further plays a central role in mediating inflammatory responses and regulating the expression of adhesion molecules and immune cell infiltration [49]. In the present study, we found that NADD notably suppressed the TLR4/NF-κB ( Figure 5) and NLRP3/Caspase-1 pathways ( Figure 6), indicating that NADD may inhibit these two pathways by blocking LPS-TLR4 binding. However, we only explored the binding of NADD to the TLR4 receptor, the binding of other receptors of LPS to NADD and other pathways mediated by NADD need to be further investigated to fully understand the anti-neuroinflammatory effect of NADD.

NADD possesses potential anti-neuroinflammatory activity in LPS-stimulated BV-2 cells. In the present study, we also used morphological observation and inflammatory signal detection to confirm the potential anti-neuroinflammatory activity of NADD *in vitro* and *in vivo*, and revealed that the TLR4/NF-κB and NLRP3/Caspase-1 pathways are the potential mechanisms of NADD’s anti-neuroinflammation activity. In addition, we identified TLR4-MD2 as the direct binding target of NADD by using SPR and molecular docking approaches. Our findings suggest that NADD is a potent inhibitor of TLR4 and NADD may have the potential to be used to treat neurodegenerative disorders.
